# Effects of *Trichoderma asperellum* 6S-2 on Apple Tree Growth and Replanted Soil Microbial Environment

**DOI:** 10.3390/jof8010063

**Published:** 2022-01-07

**Authors:** Haiyan Wang, Rong Zhang, Yunfei Mao, Weitao Jiang, Xuesen Chen, Xiang Shen, Chengmiao Yin, Zhiquan Mao

**Affiliations:** State Key Laboratory of Crop Biology, College of Horticultural Science and Engineering, Shandong Agricultural University, Tai’an 271018, China; 2020010070@sdau.edu.cn (H.W.); 2019110243@sdau.edu.cn (R.Z.); 2018010064@sdau.edu.cn (Y.M.); 2018110228@sdau.edu.cn (W.J.); chenxs@sdau.edu.cn (X.C.); shenx@sdau.edu.cn (X.S.)

**Keywords:** ARD, 6S-2 *Trichoderma* fertilizer, microbial community structure, root exudates, high-throughput sequencing

## Abstract

*Trichoderma asperellum* strain 6S-2 with biocontrol effects and potential growth-promoting properties was made into a fungal fertilizer for the prevention of apple replant disease (ARD). 6S-2 fertilizer not only promoted the growth of *Malus hupehensis* Rehd seedlings in greenhouse and pot experiments, but also increased the branch elongation growth of young apple trees. The soil microbial community structure changed significantly after the application of 6S-2 fertilizer: the relative abundance of *Trichoderma* increased significantly, the relative abundance of *Fusarium* (especially the gene copy numbers of four *Fusarium* species) and *Cryptococcus* decreased, and the relative abundance of *Bacillus* and *Streptomyces* increased. The bacteria/fungi and soil enzyme activities increased significantly after the application of 6S-2 fertilizer. The relative contents of alkenes, ethyl ethers, and citrullines increased in root exudates of *M. hupehensis* Rehd treated with 6S-2 fertilizer and were positively correlated with the abundance of *Trichoderma*. The relative contents of aldehydes, nitriles, and naphthalenes decreased, and they were positively correlated with the relative abundance of *Fusarium*. In addition, levels of ammonium nitrogen (NH_4_-N), nitrate nitrogen (NO_3_-N), available phosphorus (AP), available potassium (AK), organic matter (SOM), and pH in rhizosphere soil were also significantly related to changes in the microbial community structure. In summary, the application of 6S-2 fertilizer was effective in alleviating some aspects of ARD by promoting plant growth and optimizing the soil microbial community structure.

## 1. Introduction

Apple replant disease (ARD) is a common occurrence in apple-growing regions [1,2]. Both biotic and abiotic factors can cause ARD, but an imbalance in the soil microbial community structure is considered to play a major role [3,4]. Some studies have shown that *Fusarium* is one of the key causes of ARD in China [5,6]. The specialized *Fusarium proliferatum* f. sp. *malus domestica* MR5 (MW600437.1), which is associated with ARD in China, was recently screened and identified in our laboratory; it has been shown to cause serious damage to the apple root system (in review). With the elimination of broad-spectrum chemical fumigants, green and sustainable biological control measures have begun to emerge [4,7,8,9]. *Trichoderma*, *Bacillus*, and *Pseudomonas* have been made into microbial fertilizers that exhibit broad-spectrum antagonism against pathogens. They are also used to compensate for the deterioration in soil physical and chemical properties caused by the excessive application of chemical fertilizers [10,11,12].

Compared with other species used to make microbial fertilizer, *Trichoderma* has exhibited a greater tolerance to environmental conditions [10,13,14]. To date, *T. harzianum*, *T. atroviride*, and *T. virens* are the most commonly used *Trichoderma* species [15,16]. Recent studies have shown that *T. asperellum* can not only better prevent plant diseases but can also promote crop growth [17,18]. *T. asperellum* has been used for the biological control of diseases caused by *F. verticillioides* [18], and the application of *T. asperellum* strains reduced the disease severity of *F. oxysporum* f. sp. *lycopersici* in tomato stems and promoted tomato plant growth [17]. *T. asperellum* inoculation has shown potential as an alternative to synthetic fungicide application for the protection of onion from infection by *Stemphylium vesicarium* [19]. The soil application of *T. asperellum* GDFS1009 granules promoted the growth of maize and inhibited the mycelial growth of *F. graminearum* by approximately 60% [20]. *T. asperellum* GDFS1009 degrades fungal cells by secreting chitinase, glucanase, and protease and can parasitize harmful fungi; it also produces polyketides, alkanes, and other antifungal secondary metabolites and peptides that inhibit the growth of pathogens [21]. Xylanase secreted by *T. asperellum* ACCC30536 can stimulate the systemic resistance of host plants against pathogenic fungi and promote plant growth [22].

Based on these previous findings, *T. asperellum* shows great potential for use in sustainable agriculture. However, domestic biocontrol agents produced from *T. asperellum* are currently used primarily for the prevention and control of annual crop diseases [23,24], and there have been relatively few studies on its use for ARD. In particular, its effects on the microbial community structure in the apple rhizosphere have not previously been reported. In earlier research, we isolated an apple root endophyte designated strain 6S-2 and identified it as *T. asperellum*. 6S-2 was shown to be an efficient biocontrol agent against ARD in China and had potential plant-growth-promoting activity [25]. The aims of the present study were therefore (i) to characterize the effects of 6S-2 fertilizer application on the growth of apple plants in greenhouse, pot, and field experiments; (ii) to analyze changes in the soil microbial community structure after 6S-2 fertilizer application (especially the abundances of *Trichoderma* and harmful *Fusarium* species); (iii) to document changes in root exudates of *M. hupehensis* Rehd seedlings after 6S-2 fertilizer application and the relationship between these exudates and the soil microbial community structure; and (iv) to gain insight into the mechanisms by which 6S-2 fertilizer alleviates ARD.

## 2. Materials and Methods

### 2.1. Experimental Sites

The soils for the greenhouse and pot experiments were obtained from a 35-year-old apple orchard in Manzhuang (36.04° N, 117.11° E), Tai’an City, Shandong Province, China. The field experiment was performed at three sites in major apple-producing areas of Shandong: Laizhou (37.07° N, 119.82° E), Qixia (37.34° N, 120.85° E), and Yiyuan (36.19° N, 118.17° E). The physical and chemical properties of the soils are shown in Supplementary Table S1.

### 2.2. Production of 6S-2 Trichoderma Fertilizer

6S-2 was cultivated on PDA medium at 28 °C until the spores had grown for approximately 6 days; 10 mL of sterile water was then added to the plate, and the spores were gently scraped with a coating rod to make a spore suspension. After removing excess hyphae by filtering through four layers of sterilized lens-cleaning paper, we calculated the spore concentration under a microscope using a hemacytometer. Sterile water was used to dilute the spore solution to a concentration of 3.05 × 10^7^ CFU/mL. The spores were then expanded using the shallow plate fermentation method [26]. Four hundred grams of sterile medium (wheat bran and corn flour in a 4:1 volume ratio with 45% sterile water added) was placed in a shallow dish (30 cm × 20 cm × 5 cm), and 2% 6S-2 spore solution was added. The dish was covered with sterilized double gauze, incubated at 28 °C, and turned once every 2 d. After 10 days of fermentation, the culture was air-dried, pulverized, and sieved to obtain *T. asperellum* 6S-2 spore powder. The concentration of the resulting spore powder was 9.5 × 10^8^ CFU/mL. The 6S-2 spore powder was mixed with the blank fertilizer carrier (high-temperature sterilized, fully decomposed cow dung) at a ratio of 5%, and the water content was kept at 30% to promote natural fermentation. The concentration of spores was measured every day until it reached 10^10^ CFU/mL. Finally, the spore concentration of 6S-2 fertilizer was 2.1 × 10^10^ CFU/mL, and it was mixed with replanted soil at a volume ratio of 1%. In the blank carrier, the available nitrogen content was 0.36 mg/g, the available phosphorus content was 1.49 mg/g, and the available potassium content was 1.03 mg/g.

### 2.3. Experimental Design

Replanted soil was used for the controls, which were denoted GR (greenhouse), PR (pot), LR (Laizhou), QR (Qixia), and YR (Yiyuan). Treatments that received replanted soil with blank fertilizer carrier were denoted GC, PC, LC, QC, and YC, and treatments that received replanted soil with 6S-2 fertilizer were denoted GT, PT, LT, QT, and YT.

In mid-January 2021, *M. hupehensis* Rehd seeds were stratified at 4 °C for approximately 45 days until they became white. The seeds were then sown into nursery substrate in March 2021, and seedlings were selected when they had grown 5–6 true leaves in late April. Seedlings with no diseases or insect pests and uniform growth were selected for use in the subsequent experiments. Some seedlings were transplanted into white plastic pots (15 cm × 9 cm × 11.5 cm) that contained 4.0 kg of soil (amended with or without the treatments above) for the greenhouse experiment, and others were transplanted into clay basin pots (42 cm × 38 cm × 32 cm) that contained 13.5 kg of soil (with or without treatments) for the outdoor pot experiments. Each treatment was replicated 20 times, pots were randomly arranged, and all plants received the same management. For the field experiments, two-year-old grafted apple trees (‘T337’ rootstock and ‘Yanfu No.3’ scion) were planted at the three field sites with different soil treatments in early March 2021. 

Samples were harvested from the greenhouse experiment in early July 2021; samples were harvested from the pot experiment in the middle of July, August, and September in 2021; and samples were harvested from the field experiment in late October 2021. When sampling, five samples were obtained from the upper soil layer around each tree, mixed together, passed through a 2 mm sieve, and separated into three parts. One was stored in a refrigerator at 4 °C and used to determine the numbers of culturable microorganisms. The second was naturally air-dried and used to measure soil enzyme activities. The third was quickly placed in liquid nitrogen, returned to the laboratory, stored at −80 °C, and used for DNA extraction and RT-qPCR analysis. DNA samples from the greenhouse experiments were sent for high-throughput sequencing.

All measurements were performed using three biological replicates from each treatment, and three technical replicates were performed per biological replicate. 

### 2.4. Measurement Indices

#### 2.4.1. Plant Related Indicators

The plant heights, stem diameters, and branch lengths were measured in the field experiment using a tower ruler, vernier caliper, and tape measure, respectively; the shoot numbers were also counted. Plant heights and stem diameters of *M. hupehensis* Rehd seedlings were measured using a meter ruler and vernier caliper, respectively. Dry and fresh weights were measured with an electronic balance. Images of roots were obtained with a Scan Maker i800 Plus scanner (Microtek. Shanghai, China), and various root system parameters were measured using an LA-S plant image analyzer (Hengmei Electronic Technology, Weifang, China).

#### 2.4.2. Soil-Related Indicators

Culturable bacteria, fungi, and actinomycetes in the soil were counted using the dilution plate method [27]. The activities of urease, sucrase, neutral phosphatase, and catalase in the soil were determined using the method of Yang and Wu [28]. Total DNA was extracted from soil using an E.Z.N.A. soil DNA kit (Omega Bio-tek, Norcross, GA, USA), and the gene copy numbers of four *Fusarium* species (*F. oxysporum*, *F. proliferatum*, *F. solani*, and *F. moniliforme*) in the soil were determined using a CFX96TM Thermal Cycler (Bio-Rad, Beijing, China) [29]. The analysis of the bacterial 16S rRNA gene and the fungal ITS region was performed on the Illumina MiSeq platform (www.i-sanger.com, accessed on 25 November 2021). The sequences of the 16S rRNA primers were 338F (5′-ACTCCTACGGGAGGCAGCAG-3′) and 806R (5′-GGACTACHVGGGTWTCTAAT-3′) [30]; the sequences of the ITS primers were ITS1F (5′-CTTGGTCATTTAGAGGAAGTAA-3′) and ITS2R (5′-GCTGCGTTCTTCATCGATGC-3′) [31]. 

#### 2.4.3. Bioinformatics Analysis

Three soil samples from each treatment in the greenhouse experiment were used for DNA extraction, PCR, and sequencing on the Illumina platform. Raw fastq files were demultiplexed and quality-filtered with Uparse (version 7.0.1090 http://drive5.com/uparse/, accessed on 30 November 2021). The 300 bp reads were truncated at any site that received an average quality score of <20 over a 50 bp sliding window, and truncated reads shorter than 50 bp were discarded. Exact barcode matching was required; reads with a 2-nucleotide mismatch in primer matching and reads that contained ambiguous characters were removed. Only sequences that overlapped by more than 10 bp were assembled according to their overlapping sequence. Operational taxonomic units (OTUs) were clustered with a 97% similarity cutoff. A total of 534,996 high-quality 16S rDNA sequences and 508,218 high-quality ITS sequences were obtained from the nine soil samples (three samples each from the three soil treatments). These sequences were distributed among 4047 bacterial OTUs and 1004 fungal OTUs. The rarefaction curves showed that the sequencing work was relatively comprehensive in covering bacterial and fungal diversity, as the curves tended to approach saturation (Supplementary Figure S1a,c). The Shannon–Wiener curve indicated that the dataset from the diversity analysis was large enough to reflect the full microbial diversity information in the samples (Supplementary Figure S1b,d).

#### 2.4.4. Determination of Root Exudate Composition

We optimized the methods of Liu et al. [32] and Wang [6] to collect and analyze root exudates from *M. hupehensis* Rehd seedlings. Three replicate seedlings from each treatment from greenhouse experiment were removed from their containers in early July 2021, and surface impurities were washed from their root systems in running water. The roots were then rinsed with sterile water, and care was taken not to damage them. Each plant was placed into a glass flask filled with 1 L sterile water, and all plants were placed in a growth chamber for 48 h (16-h light/8-h dark) at 25 ± 5 °C with gentle shaking (50 rpm). Plants were then removed from their flasks, and the resulting exudate solution was filtered using a 0.45-μm filter (Millipore) and extracted 3 times with ethyl acetate at a volume ratio of 1:1. The three extracts were combined and concentrated to 5 mL under reduced pressure at 30 °C. After passing again through a 0.45 μm organic membrane, the crude extract was used for GC-MS analysis.

Chromatography was performed using an Rtx-5MS column (30 m × 0.32 mm × 0.25 μm) with a column oven temperature of 50 °C and an injection port temperature of 230 °C. The sample was injected with a split ratio of 10.0, and the injection volume was 1 μL. High purity He was used as the carrier gas at a pressure of 117.6 kPa and a column flow rate of 2.4 mL/min. The temperature program was as follows: 50 °C for 2 min, increased to 250 °C at 6 °C/min, and held for 10 min. The mass spectrometry conditions included Q3 scan acquisition mode, relative value EMV mode, a full scan acquisition mass range of 45–550 amu, ion source EI of 70 eV, and a temperature of 200 °C. The experimental results were compared with spectra at the NIST 17 database, and the peak area normalization method was used to express the relative content of each metabolite as the ratio of its peak area relative to the total peak area. The triple quadrupole gas chromatograph–mass spectrometer (GCMS-TQ8040 NX) and peak processing software were all from Shimadzu (Beijing, China). The retention time was used for qualitative identification, and the peak areas of external standards were used for quantification.

### 2.5. Statistical Analysis

A heatmap of microbial abundance data was constructed using the gplot package in R. The edge-weighted spring-embedding algorithm pulled together similar related properties and systems with similar structures. Networkx was used to calculate the node degree distribution, network diameter, average shortest path, node connectivity (degree), closeness centrality, betweenness centrality, and other network attributes to obtain relevant information within or between groups of species and samples. Principal coordinate analysis (PCoA) was performed based on the Bray–Curtis distance matrix calculated from the genus information of each sample. Hierarchical clustering analysis at the genus level was performed using the UPGMA (unweighted pair group method with arithmetic mean) algorithm based on Bray–Curtis distances generated by mothur. LEfSe analysis was also performed; this metagenomic approach uses linear discriminant analysis to determine the taxa that were most likely to explain differences among treatments. Distance-based redundancy analysis (db-RDA), a constrained extension of PCoA, was used to show the relationships of environmental factors and treatments to microbial community structure and was performed with the capscale function in the vegan R package. The established biological correlation network was analyzed based on an understanding of graph theory. The data were digitized using Microsoft Excel 2010. Analysis of variance was performed using IBM SPSS 19.0, and Duncan’s new complex range method was used to assess the significance of differences. Data were presented as mean ± SE (standard error). Graph Pad Prism 7 was used to construct the figures. 

To facilitate subsequent descriptions, the names of the experimental treatments were simplified and abbreviated as follows: GR (control replant soil in the greenhouse experiment); GC (replant soil with blank carrier in the greenhouse experiment); GT (replant soil with 6S-2 fertilizer in the greenhouse experiment); PR (control replant soil in the pot experiment); PC (replant soil with blank carrier in the pot experiment); PT (replant soil with 6S-2 fertilizer in the pot experiment); LR (control replant soil in Laizhou); LC (replant soil with blank carrier in Laizhou); LT (replant soil with 6S-2 fertilizer in Laizhou); QR (control replant soil in Qixia); QC (replant soil with blank carrier in Qixia); QT (replant soil with 6S-2 fertilizer in Qixia); YR (control replant soil in Yiyuan); YC (replant soil with blank carrier in Yiyuan); YT (replant soil with 6S-2 fertilizer in Yiyuan).

## 3. Results

### 3.1. Growth of M. hupehensis Rehd Seedlings and Young Apple Trees

**Figure 1:**
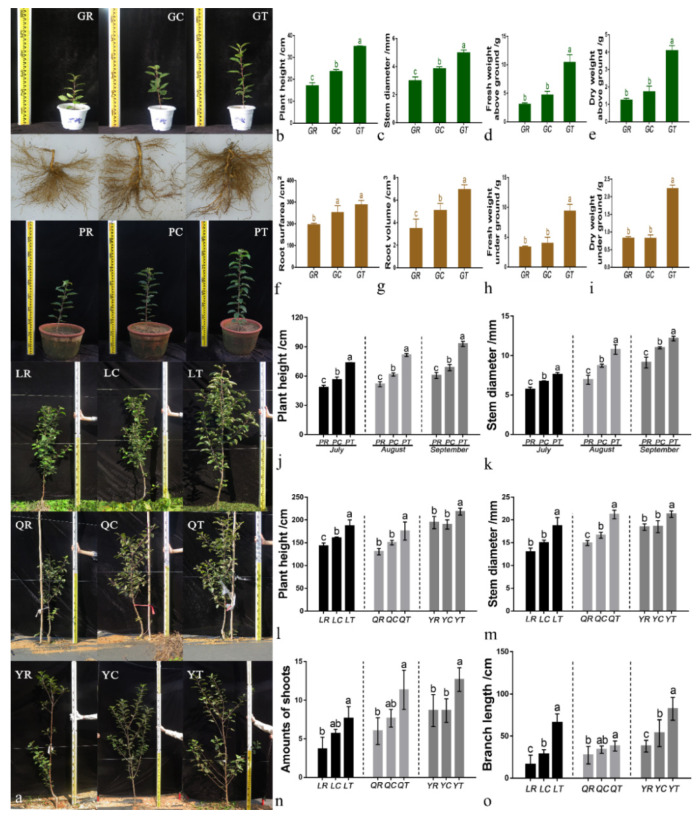
Phenotypes of plants that received different soil treatments in greenhouse, pot, and field experiments (**a**). Plant height (**b**), stem diameter (**c**), aboveground fresh weight (**d**), aboveground dry weight (**e**), root surface area (**f**), root volume (**g**), aboveground fresh weight (**h**), and belowground dry weight (**i**) of *M. hupehensis* Rehd seedlings in the greenhouse experiment. Plant height (**j**) and stem diameter (**k**) of *M. hupehensis* Rehd grown in pots from July to September 2021. Plant height (**l**), stem diameter (**m**), branch number (**n**), and branch length (**o**) of young apple trees in the field experiment in October 2021. GR, control replant soil in the greenhouse experiment; GC, replant soil with blank carrier in the greenhouse experiment; GT, replant soil with 6S-2 fertilizer in the greenhouse experiment; PR, control replant soil in the pot experiment; PC, replant soil with blank carrier in the pot experiment; PT, replant soil with 6S-2 fertilizer in the pot experiment; LR, control replant soil in Laizhou; LC, replant soil with blank carrier in Laizhou; LT, replant soil with 6S-2 fertilizer in Laizhou; QR, control replant soil in Qixia; QC, replant soil with blank carrier in Qixia; QT, replant soil with 6S-2 fertilizer in Qixia; YR, control replant soil in Yiyuan; YC, replant soil with blank carrier in Yiyuan; YT, replant soil with 6S-2 fertilizer in Yiyuan. The displayed data were presented as mean ± SE (standard error). Different lowercase letters in the same column indicate a significant difference at *p* ≤ 0.05 level by Duncan’s new multiple range test. The same treatment names and statistical tests were used in subsequent figures.

The application of 6S-2 fertilizer promoted the growth of *M. hupehensis* Rehd seedlings and two-year-old grafted apple trees (Figure 1). There was a significant difference in plant height between control plants grown in replant soil and plants grown in replant soil with 6S-2 fertilizer (Figure 1b,j,l). Under greenhouse conditions, the root dry and fresh weights of *M. hupehensis* Rehd seedlings grown with 6S-2 fertilizer were 1.82- and 1.70-fold higher than those of the control, and 1.37- and 1.66-fold higher than those of blank carrier-treated plants (Figure 1h–i). Under field conditions, the application of 6S-2 fertilizer significantly increased the number of branches and branch elongation of young apple trees (Figure 1n,o).

### 3.2. Analysis of Soil Microbial Community Composition at the Genus Level

**Figure 2:**
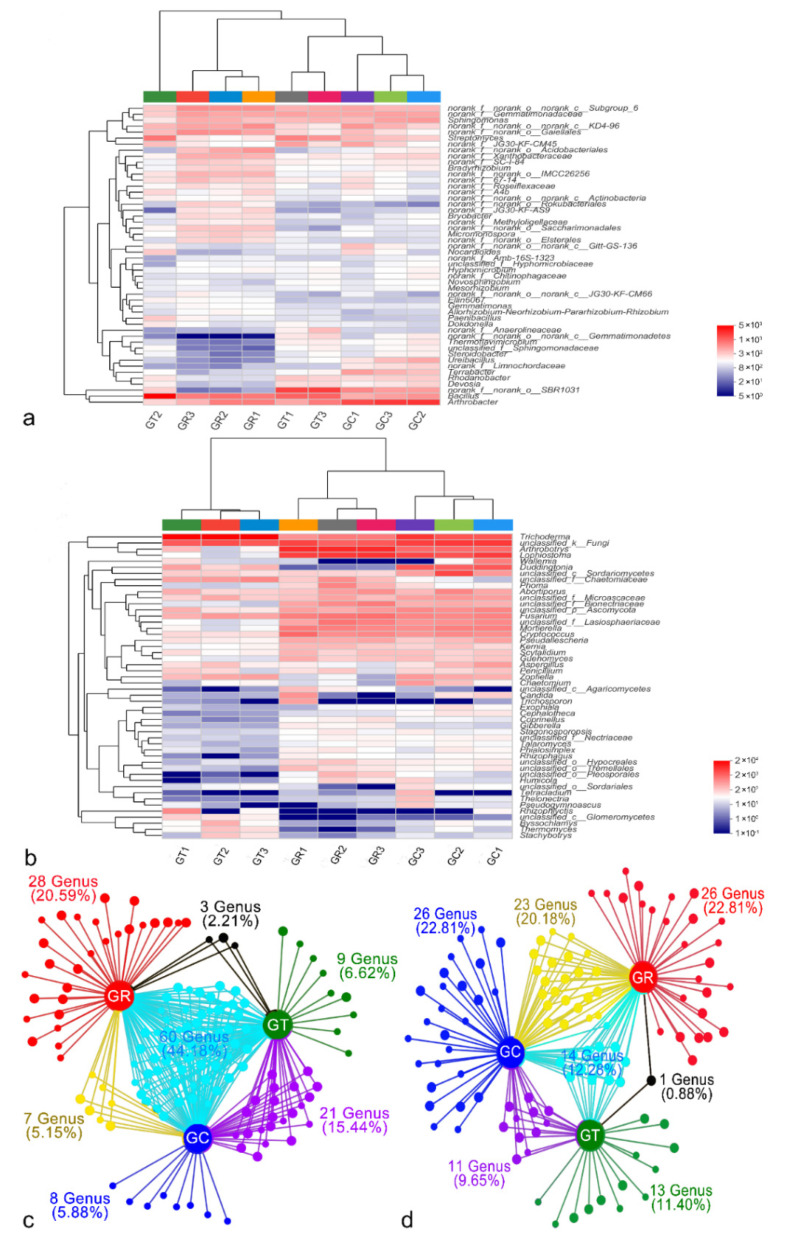
Soil microbial community composition. Heatmaps showing differences in community structure of bacteria (**a**) and fungi (**b**) at the genus level. Collinearity network analysis in bacteria (**c**) and fungi (**d**) at the genus level based on species abundances greater than 50; the node sizes represent the relative abundance (square root) of the genus in the data set, and the edges represent the association patterns of individual genera with different treatments. The red nodes represent genera related to GR, the blue nodes represent genera related to GC, the green nodes represent genera related to GT, the yellow nodes represent genera related to GR and GC, the black nodes represent genera related to GR and GT, the purple nodes represent genera related to GT and GC, and the cyan nodes represent genera related to GR, GC, and GT.

The top 50 dominant bacterial and fungal genera across all samples were used to construct an abundance heatmap, and differences in the abundances of soil bacteria and fungi were apparent after the application of 6S-2 fertilizer (Figure 2). The fungal compositions of GT and GC were not clustered on the same branch, indicating that their fungal communities differed significantly (Figure 2b). At the genus level (after removal of unidentified strains), *Bacillus*, *Arthrobacter*, *Streptomyces*, *Sphingomonas*, and *Terrabacter* were the dominant bacterial species (Figure 2a; Supplementary Figure S2a), and *Trichoderma*, *Arthrobotrys*, *Lophiostoma*, *Duddingtonia*, and *Fusarium* were the dominant fungal species (Figure 2b; Supplementary Figure S2b). After the application of 6S-2 fertilizer, the relative abundances of *Bacillus* and *Trichoderma* increased to 6.91% and 70.48%, respectively, but the relative abundance of *Fusarium* decreased significantly (Figure 2; Supplementary Figure S3). Collinearity network analysis at the genus level showed that the GR and GT groups shared the smallest proportion of specific bacteria and fungi, accounting for only 2.21% and 0.88%, respectively (Figure 2c,d).

### 3.3. Analysis of Culturable Microorganisms and Real-Time Fluorescence Quantification of Four Fusarium Species

**Figure 3:**
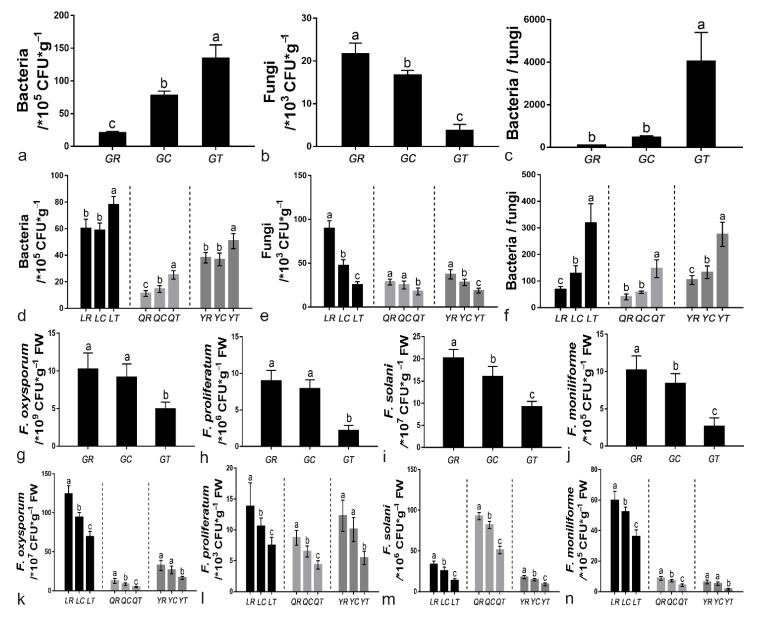
The number of culturable bacteria in the greenhouse experiment (**a**) and field experiment (**d**), and the number of culturable fungi in the greenhouse experiment (**b**) and field experiment (**e**). The ratio of bacteria to fungi in the greenhouse experiment (**c**) and field experiment (**f**). Real-time fluorescence quantification of *F. oxysporum* in the greenhouse experiment (**g**) and field experiment (**k**), *F. proliferatum* in the greenhouse experiment (**h**) and field experiment (**l**), *F. solani* in the greenhouse experiment (**i**) and field experiment (**m**), and *F. moniliforme* in the greenhouse experiment (**j**) and field experiment (**n**). Different lowercase letters (a,b,c) in the same column indicate a significant difference at *p* ≤ 0.05 level by Duncan’s new multiple range test.

Application of 6S-2 fertilizer increased the number of culturable bacteria and decreased the number of culturable fungi relative to the control treatment by 71.88%, 37.43%, and 50.00% in Laizhou, Qixia, and Yiyuan, respectively, and these differences were statistically significant (Figure 3a,b,d,g). As a result, the 6S-2 fertilizer caused a marked increase in the bacteria/fungi ratio (Figure 3c,g). In the greenhouse and field environments, there was little difference in the relative abundance of four *Fusarium* species between the control treatment and the blank carrier treatment. By contrast, the gene copy numbers of *F. oxysporum*, *F. proliferatum*, *F. solani*, and *F. moniliforme* declined to various extents (39.80–73.85%) in the 6S-2 fertilizer treatment (Figure 3e–i,k–n). Similar results were observed in the pot experiment (Supplementary Figure S4).

### 3.4. Differences in Microbial Species among Treatments

**Figure 4:**
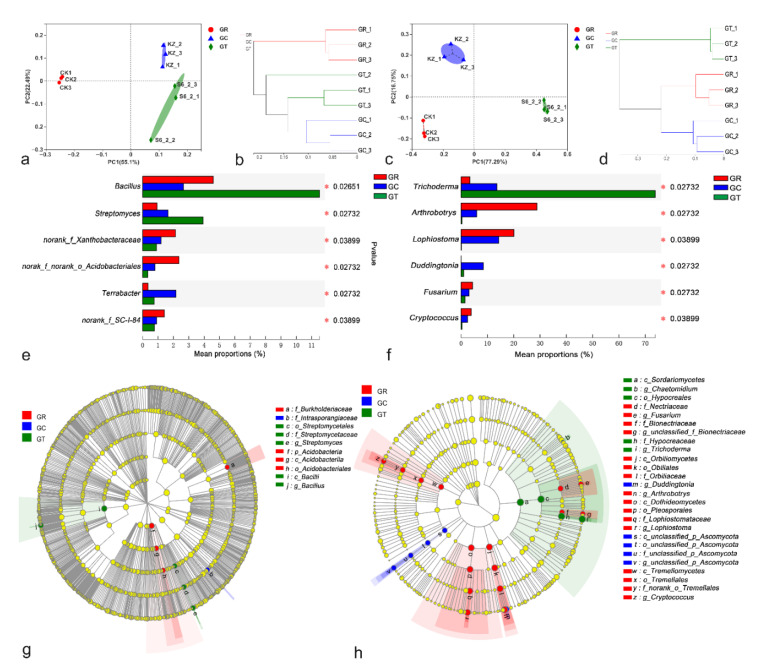
Principal coordinate analysis (PCoA) of the Bray–Curtis distance matrix at the genus level for the bacterial (**a**) and fungal (**c**) communities. Circles, triangles, and diamonds represent the GR, GC, and GT samples. Hierarchical clustering analysis at the genus level for bacteria (**b**) and fungi (**d**). Statistical comparisons of the relative abundances of bacteria (**e**) and fungi (**f**) at the genus level in different groups were performed using the Kruskal–Wallis test. Cladograms showing the phylogenetic distribution of the bacterial (**g**) and fungal (**h**) lineages associated with the three treatments were generated by LEfSe analysis. Different colored regions represent different groups (red, GR; blue, GC; green, GT). Circles indicate phylogenetic levels from phylum to genus. The diameter of each circle is proportional to the abundance of the group.

A PCoA of Bray–Curtis distance matrix distances between samples revealed differences in their bacterial and fungal communities. The first two principal component scores accounted for 77.59% of the total variation in bacteria (Figure 4a) and for 94.04% of the total variation in fungi (Figure 4c), suggesting that the application of 6S-2 fertilizers may be one of the important factors driving changes in the microbial community structure. Based on their different microbial communities, samples were clustered into two groups, one of which corresponded to GR in bacteria (Figure 4b) and the other to GT in fungi (Figure 4d). Overall, the results demonstrated a clear division between GR and GT. At the genus level, the top six genera of both bacteria and fungi differed significantly among the three groups. *Bacillus*, *Streptomyces*, and *Trichoderma* were more abundant after 6S-2 fertilizer application, whereas *Arthrobotrys*, *Lophiostoma*, and *Fusarium* declined markedly (Figure 4e,f). Groups were displayed in cladograms, and LDA scores of 4 or greater were confirmed by LEfSe (Figure 4g,h; Supplementary Figure S5). Two groups of bacteria and five groups of fungi were significantly enriched in GR: *Acidobacteriales* (from phylum to order), *Burkholderiaceae* (family), *Cryptococcus* (from class to genus), *Lophiostoma* (from class to genus), *Arthrobotrys* (from class to genus), *Fusarium* (from family to genus), and *Bionectriaceae* (from family to genus). Fewer microbes were significantly enriched in GC. Two groups of bacteria and two groups of fungi were significantly enriched in GT: *Streptomyces* (from order to genus), *Bacillus* (genus and its class Bacilli), *Chaetomidium* (genus, the class Sordariomycetes and the order Hypocreales), and *Trichoderma* (from family to genus). These results showed that there was a significant difference in the composition of the soil microbial community between GR and GT.

### 3.5. Relationships between Microbial Community Structure and Environmental Factors

**Figure 5:**
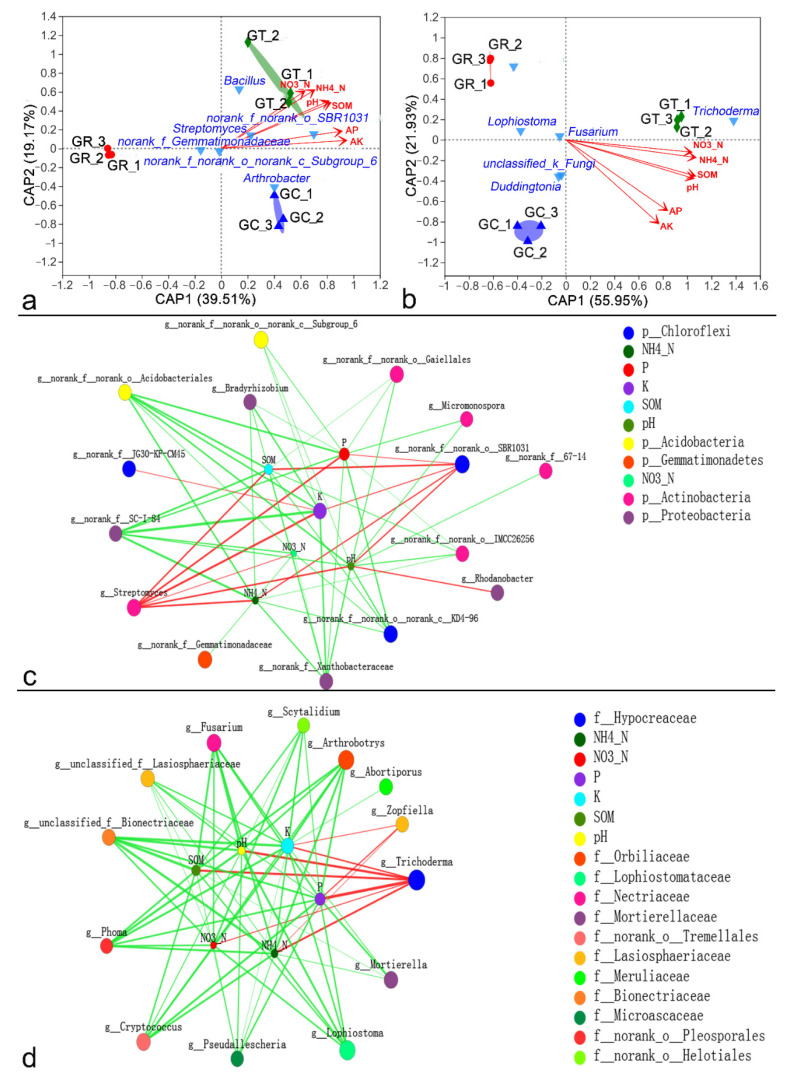
Distance-based redundancy analysis (db-RDA) plot showing the relationships of environmental factors and treatments with bacterial (**a**) and fungal (**b**) community structure. The values on axes 1 and 2 are the percentages explained by each axis. Circles, upright triangles, and diamonds represent the GR, GC, and GT samples. The inverted triangles represent the top six microbial species in terms of abundance, and the red arrows represent environmental factors. Two-way correlation network analysis between environmental factors and the top twenty bacterial (**c**) and fungal (**d**) genera. The size of the node is proportional to the genus abundance and environmental factors. Node color corresponds to the family taxonomic classification. Edge colors represent positive (green) and negative (red) correlations, and the edge thickness is equivalent to the correlation magnitude.

Db-RDA revealed that the soil microbial community structure was influenced by environmental factors, including ammonium nitrogen (NH_4_-N), nitrate nitrogen (NO_3_-N), available phosphorus (AP), available potassium (AK), soil organic matter (SOM), and pH in rhizosphere soil. All these factors significantly affected the bacterial and fungal community structure (*p* *≤* 0.05) (Figure 5a,b; Supplementary Table S2). The GT groups were positively correlated with environmental factors, but the GR groups were negatively correlated with them (Figure 5a,b). Two-way correlation network analysis showed that bacterial genera and fungal genera had different relationships with environmental factors (Figure 5c,d). *Streptomyces* and *Trichoderma* showed significant positive correlations with all environmental factors, whereas *Fusarium*, *Lophiostoma*, *Arthrobotrys*, and *Cryptococcus* showed a highly significant negative correlation with NH_4_-N, NO_3_-N, AP, AK, SOM, and pH (Supplementary Figure S6). There were antagonistic or synergistic effects between different microbial genera, and the top six most abundant bacterial species showed fewer associations. *Fusarium*, *Lophiostoma*, *Arthrobotrys*, and *Cryptococcus* showed synergistic relationships with one another but showed strong antagonism toward *Trichoderma* (Supplementary Figure S7).

### 3.6. Soil Enzyme Activities

**Figure 6:**
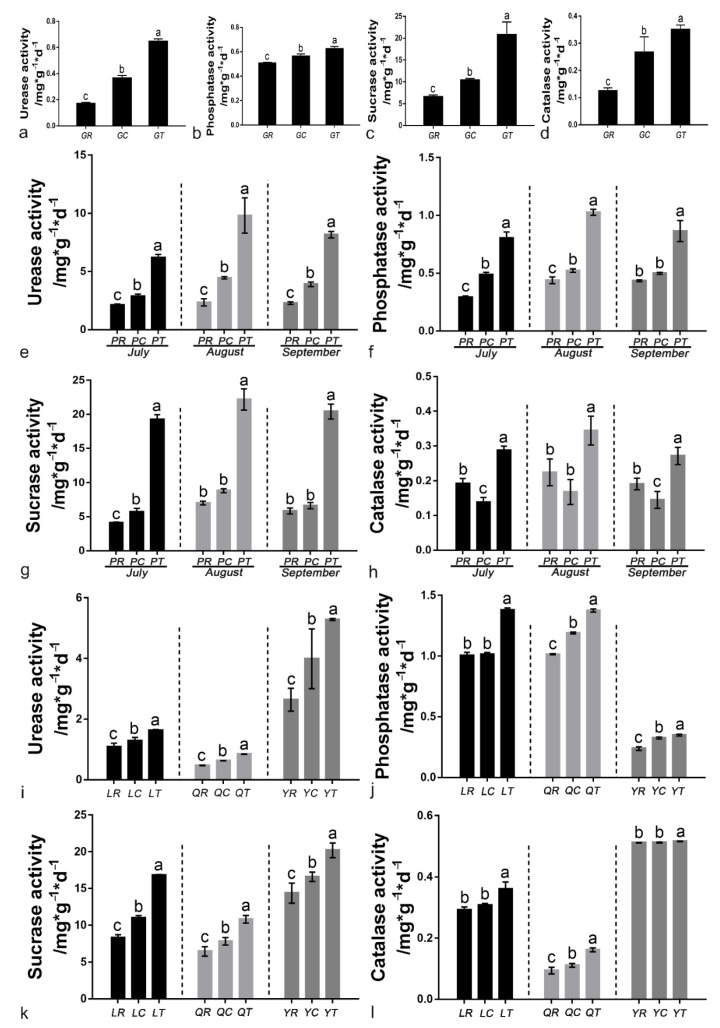
Soil urease activity in the greenhouse experiment (**a**), pot experiment (**e**), and field experiment (**i**); soil phosphatase activity in the greenhouse experiment (**b**), pot experiment (**f**), and field experiment (**j**); soil sucrase activity in the greenhouse experiment (**c**), pot experiment (**g**), and field experiment (**k**); and soil catalase activity in the greenhouse experiment (**d**), pot experiment (**h**), and field experiment (**l**). Different lowercase letters (a,b,c) in the same column indicate a significant difference at *p* ≤ 0.05 level by Duncan’s new multiple range test.

Application of 6S-2 fertilizer increased the activities of urease, phosphatase, invertase, and catalase to various degrees in the greenhouse, pot, and field experiments, and there were significant differences between the GR and GT treatments (Figure 6). In the pot experiment, the activities of the four soil enzymes peaked in August (Figure 6e–h). Compared with control replant soil, the urease activity in 6S-2-treated soil increased by 49.99%, 78.82%, and 100.31% in Laizhou, Qixia, and Yiyuan, respectively; the sucrase activity increased by 101.83%, 67.00%, and 40.46%; the phosphatase activity increased by 37.12%, 35.59%, and 46.99%; and the catalase activity increased by 49.99%, 78.82%, and 100.31% (Figure 6i–l).

### 3.7. Root Exudate Components and Correlation Analysis with the Microbial Community

**Figure 7:**
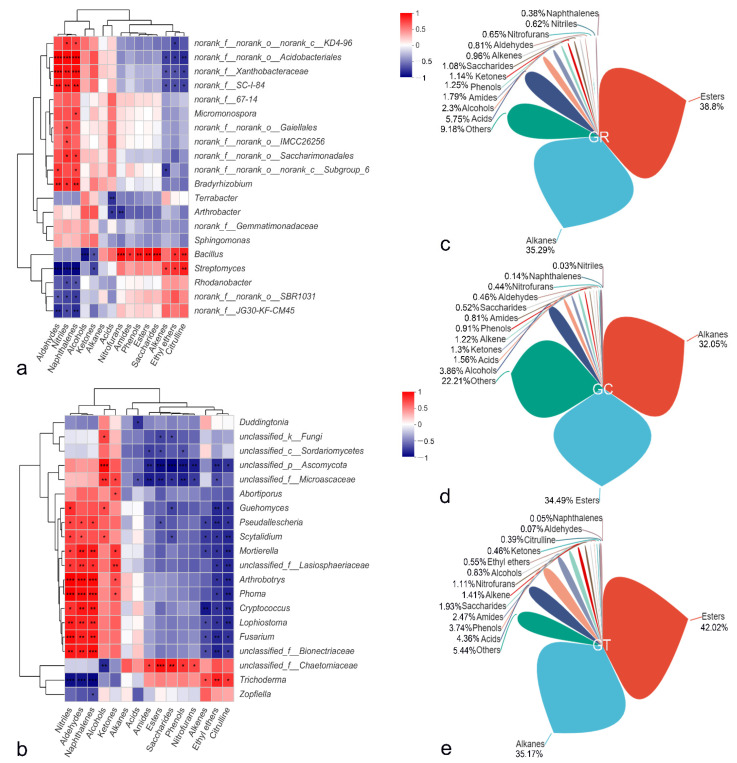
Correlation heatmap of the top twenty bacterial (**a**) and fungal (**b**) genera with root exudates. The x and y axes are root exudates and genera. The legend shows the color range of the R values. Pie charts show the percentages of different root exudate components in GR (**c**), GC (**d**) and GT (**e**). Different colored petals represent different components. GR, control replant soil in the greenhouse experiment; GC, replant soil with blank carrier in the greenhouse experiment; GT, replant soil with 6S-2 fertilizer in the greenhouse experiment. * *p* ≤ 0.05, ** *p* ≤ 0.01, *** *p* ≤ 0.001.

Root exudates of plants from the GR, GC, and GT treatments all contained esters, alkanes, acids, alcohols, amides, phenols, ketones, saccharides, alkenes, aldehydes, nitrofurans, and naphthalenes. Esters and alkanes were the main components. Ethyl ethers and citrulline were present only in the GT group, but nitriles were absent (Figure 7c–e). The peak area of dibutyl phthalate was as high as 41.82% in the GT treatment, 7.65% higher than that of the control. The components with peak areas greater than 0.5% in root exudates of the three treatment groups are shown in Supplementary Table S3, and the associated spectra are provided in Supplementary Figure S8. Alkanes, ethyl ethers, and citrullines were significantly positively correlated with *Streptomyces* and *Trichoderma* but significantly negatively correlated with *Fusarium*, *Lophiostoma*, *Arthrobotrys*, and *Cryptococcus.* Nitrofurans, saccharides, phenols, and citrullines were significantly positively correlated with *Bacillus*, but alcohols were negatively correlated with this taxon (Figure 7a,b).

## 4. Discussion

The root system is the link between the plant and the soil. A healthy soil environment can promote the development of the root system, which, in turn, promotes the growth of plants [33]. After 6S-2 fertilizer was applied to the soil, the relative abundance of *Trichoderma* in the rhizosphere increased significantly, whereas the relative abundance of harmful fungi such as *Fusarium* decreased (Figure 2b and Figure 4e). This result suggested that 6S-2 can rapidly multiply in apple rhizosphere soil and can inhibit the propagation of pathogenic *Fusarium* [34] through direct niche competition [35]. This result may also reflect a direct interaction between 6S-2 and *Fusarium*, whereby 6S-2 exhibits antagonism, reparasitism, and bacteriolysis that reduces the relative abundance of *Fusarium* [36]. Previous work has shown that the diversity of the soil microbial community was enriched after *Trichoderma* colonization, resulting in a significant reduction in the population of *Fusarium* [37,38]. After 6S-2 fertilizer was applied to replanted apple orchards soil, the bacteria/fungi increased significantly (Figure 3c,j), demonstrating that 6S-2 fertilizer can effectively regulate the ratio of bacteria to fungi in the apple rhizosphere [39]. The soil community structure changed from a fungal type to a bacterial type; this may improve the structure and function of the soil microbial flora [39,40] and may stimulate the proliferation of beneficial bacteria such as *Bacillus* and *Streptomyces* (Figure 2a and Figure 4f). In a previous report, colony multiplication drove interactions among the soil microflora, reduced the number of harmful *Fusarium,* and effectively controlled the occurrence of soil-borne diseases [41]. *Trichoderma* has also shown a variety of positive effects on plant growth, resilience, and yield [42,43]. In the experiments reported here, the application of 6S-2 fertilizer promoted the growth of young apple trees and *M. hupehensis* Rehd seedlings (Figure 1). Changes in microbial species may also depend on the symbiotic interaction between plants and their surrounding microorganisms [42]. After the application of microbial fertilizer, biocontrol microorganisms can quickly form a “substrate–microorganism” ecosystem with the help of the carrier [44]; this process helps to regulate the soil micro-ecological environment, promotes the restoration of soil enzyme activities, and changes the ecology of the rhizosphere through its effects on the physical and chemical properties of soil microorganisms [45,46]. The new optimized soil environment can, in turn, promote the further growth and development of 6S-2 and other biocontrol microorganisms [39,47]; it can also enhance the secretion of biocontrol enzymes, IAA, and other secondary metabolites and can improve plant growth [40] and stress resistance [48].

Root exudates are the bridge between plants, soil, and microbes and play an important role in the interaction between plants and the environment [49]. The type and quantity of root exudates determine the type and quantity of rhizosphere microorganisms [41,50], and rhizosphere microorganisms in turn affect the production of root exudates [41]. Root exudates of different crops can regulate different aspects of the rhizosphere microbial community [51]. Under continuous cropping conditions, watermelon root exudates can significantly increase the number of germinated *Fusarium* spores and enhance their reproductive ability [52]. The accumulation of phloridzin and other phenolic autotoxic substances in replanted apple soil hinders apple growth [53]. In this experiment, the composition of *M. hupehensis* Rehd seedlings root exudates changed after the application of 6S-2 fertilizer (Figure 7c–e). Previous works raise the possibility that 6S-2 application may adjust the root exudate composition to promote the recruitment and aggregation of specific beneficial microorganisms [54], thereby optimizing the rhizosphere microbial community structure [55] and inducing plant resistance to pathogenic fungi such as *Fusarium* [56]. The accumulation of dibutyl phthalate and other substances has been shown to directly inhibit the growth of harmful fungi such as *Fusarium* and the germination of spores, thereby helping to limit pathogen damage [57]. The relative contents of sugars and amino acids increased in root exudates after the application of 6S-2 fertilizer. This may have provided the carbon and energy required for 6S-2 growth [58,59], enhanced the absorption and utilization of nutrients under stress conditions [58], and thus promoted plant growth. Perhaps because of the different extraction methods, detection methods, and plant species, we did not detect large amounts of sugars, fatty acids, amino acids, and other substances, in contrast to the results of previous studies [60,61]. Related methods need to be continuously optimized in follow-up research to more accurately determine the composition of root exudates [62]. The relationships between root exudates and rhizosphere soil microorganisms also require further study [60].

The effects of *Trichoderma* fertilizers were closely related to the application environment [63]. At the same application rate, 6S-2 fertilizers showed greater effects in greenhouse and pot experiments than in the field experiment. To improve its biological control effect, it will be necessary to determine which environmental conditions are most conducive to the colonization of *Trichoderma* and enhance its ability to compete with the indigenous microbial flora. It is unclear whether the changes in the soil microbial community structure and plant root exudates caused by the application of 6S-2 fertilizer will continue for a long time [61]. The duration of the fertilizer effects and whether they can directly control ARD will require additional testing [64]. Further experiments are also needed to separate and identify chemotactic substances in root exudates and to determine whether their combination with 6S-2 is more effective for the prevention and treatment of ARD. The optimal application rate, timing, and frequency for 6S-2 fertilizer are also important issues that must be considered in field production. The effects of *Trichoderma* are related not only to the species itself but also to its application method [65]. The application of solid *Trichoderma* fertilizers, liquid fungal agents, or spore suspensions have all been shown to alleviate continuous cropping obstacles to some extent [66]. Therefore, reducing the number of processing procedures, lowering production costs, extending shelf life, and optimizing the application rate are the top priorities for subsequent research.

## 5. Conclusions

The application of 6S-2 *Trichoderma* fertilizer to replanted soil promoted increases in apple biomass and increased the ratio of bacteria to fungi in the soil. In particular, it increased the relative abundance of *Trichoderma*, Bacillus, and Streptomyces and reduced the relative abundance of harmful *Fusarium*. 6S-2 fertilizer thus altered the soil microbial community structure, perhaps through its marked effects on the relative content of multiple root exudate components. Therefore, the application of 6S-2 fertilizers to replanted soil can both promote plant growth and optimize the soil microbial community structure and can help to alleviate ARD.

## Data Availability

The study did not report any data.

## Supplementary Materials

The following supporting information can be downloaded at: https://www.mdpi.com/article/10.3390/jof8010063/s1. Figure S1: Dilution curve at the OTU level. Sobs index of bacteria (a) and fungi (c); Shannon index of bacteria (b) and fungi (d); Figure S2: Species compositions of different treatments at the genus level for bacteria (a) and fungi (b); Figure S3: Circos diagrams of the relative abundance and distribution of soil bacteria (a) and fungi (b) at the genus level for different groups. The width of the bar indicates the relative abundance of the genus; Figure S4: The number of culturable bacteria (a) and fungi (b) and the bacteria/fungi (c) in the pot experiment. Real-time fluorescence quantification of *F. oxysporum* (d), *F. proliferatum* (e)*, F. solani* (f), and *F. moniliforme* (g) in the pot experiment; Figure S5: LDA scores of enriched taxa from Figure 4 (g and h). Indicator bacteria (a) and fungi (b) with LDA scores of 4 or greater in communities of the three treatment groups. GR (red), GC (blue), and GT (green); Figure S6: Correlation heatmap of the top fifty bacterial (a) and fungal (b) genera with environmental factors. The x and y axes are environmental factors and genera. The legend shows the color range of the *R* values. * *p* ≤ 0.05, ** *p* ≤ 0.01, *** *p* ≤ 0.001.; Figure S7: Single-way correlation network among the top twenty bacterial (c) and fungal (d) genera. Node size is proportional to genus abundance. Node color corresponds to family taxonomic classification. Edge colors represent positive (green) and negative (red) correlations, and the edge thickness is equivalent to the correlation value; Figure S8: The spectrum of exudates from roots of *M. hupehensis* Rehd seedlings under different treatments. The x and y axes are time and contents; Table S1: Physical and chemical properties of soils from four apple orchards; Table S2: The environmental factors used in db-RDA analysis; Table S3: Components with peak area greater than 0.5% in the root exudates of *M. hupehensis* Rehd under different treatments.
