# Supplementary material for: Effects of Trichoderma asperellum 6S-2 on Apple Tree Growth and Replanted Soil Microbial Environment
Source: J Fungi (Basel). 2022 Jan 7;8(1):63. doi: 10.3390/jof8010063 (PMC8778220; doi:10.3390/jof8010063)
Supplement: Supplementary file 1 [file jof-08-00063-s001.zip › jof-1533036-supplementary.pdf]

Journal of Fungi

MDPI

Supplementary

Table S1. Physical and chemical properties of soils from four apple orchards.

Sites	NH_4-N ($mg \cdot kg^{-1}$)	NO_3-N ($mg \cdot kg^{-1}$)	AP ($mg \cdot kg^{-1}$)	AK ($mg \cdot kg^{-1}$)	SOM ($g \cdot kg^{-1}$)	pH	Soil Texture
Manzhuang	3.27	5.95	43.65	56.32	8.16	6.98	sandy loam
Laizhou	3.96	11.55	29.42	21.78	10.52	7.26	clay loam
Qixia	1.13	12.87	18.38	20.59	11.38	5.53	sandy loam
Yiyuan	1.05	20.41	16.55	30.31	12.39	7.73	Sandy soil

Note: AP: available phosphorus; AK: available potassium; SOM: soil organic matter.

Table S2. The environmental factors used in db-RDA analysis.

	Environmental factors	CAP1	CAP2	r^2	*p*_Values
Bacteria	NH_4-N	0.7552	0.6555	0.9479	0.001
	NO_3-N	0.7245	0.6893	0.8194	0.016
	P	0.9804	0.1972	0.934	0.008
	K	0.9957	0.0921	0.9797	0.01
	SOM	0.8655	0.501	0.9919	0.002
	pH	0.8532	0.5215	0.9849	0.003
Fungi	NH_4-N	0.9871	−0.16	0.9273	0.005
	NO_3-N	0.9932	−0.1166	0.8581	0.017
	P	0.7712	−0.6365	0.9277	0.021
	K	0.6838	−0.7297	0.99	0.013
	SOM	0.9411	−0.338	0.9914	0.002
	pH	0.9492	−0.3146	0.9978	0.001

Table S3. The components with peak area greater than 0.5% in the root exudates of *M. hupehensis* under different treatments.

	Retention time	Area (%)	Possible Compounds	CAS Number	Molecular Formula
GR	18.824	34.17	1,2-Benzenedicarboxylic acid, bis(2-methylpropyl) ester	84-69-5	$C_{16}H_{22}O_4$
	23.964	2.79	Hexatriacontane	630-06-8	$C_{36}H_{74}$
	22.909	1.45	Acetic acid, octadecyl ester	822-23-1	$C_{20}H_{40}O_2$
	14.261	1.32	3-Acetoxybutyric acid	52020-45-8	$C_6H_{10}O_4$
	21.134	1.2	Benzocycloheptano[2,3,4-I,j]isoquinoline	131179-95-8	$C_{20}H_{23}NO_4$
	10.42	1.19	Caprolactam	105-60-2	$C_6H_{11}NO$
	14.887	1.1	Fluorene	86-73-7	$C_{13}H_{10}$
	19.987	1.09	Heneicosane	629-94-7	$C_{21}H_{44}$
	13.654	1.08	3-Deoxyglucose	4084-27-9	$C_6H_{10}O_5$
	22.25	1	Oxalic acid	144-62-7	$C_2H_2O_4$
	20.344	0.99	Cyclopropane, 1,1-dichloro-2,2-dimethyl-3-(2-methylpropyl)-	24577-81-9	$C_9H_{16}Cl_2$
	20.215	0.91	Cyclopropanecarboxylic acid,2-(1-trimethylsilylpropyn-3-yl), methyl ester	775-56-4	$C_{11}H_{18}O_2Si$
	20.996	0.9	Acetic acid, octadecyl ester	822-23-1	$C_{20}H_{40}O_2$
	21.207	0.89	tert-Hexadecanethiol	25360-09-2	$C_{14}H_{21}O^-.K^+$
	21.328	0.82	5,8,11,14-Eicosatetraynoic acid	1191-85-1	$C_{20}H_{24}O_2$
	24.425	0.8	Tetrapentacontane, 1,54-dibromo-	852228-22-9	$C_{54}H_{108}Br_2$

	21.384	0.77	Cyclopropane, 1,1-dichloro-2,2,3-triethyl-	24551-90-4	$C_9H_{16}Cl_2$
	23.243	0.61	Phenol, 2,2'-methylenebis[6-(1,1-dimethylethyl)-4-methyl-	93803-63-5	$C_{27}H_{40}O_2$
	21.255	0.6	s-Triazole-3-carboxaldehyde, 5-(p-chlorophenyl)-	26899-27-4	$C_9H_6ClN_3O$
	29.445	0.6	cis-11-Eicosenamide	10436-08-5	$C_{20}H_{39}NO$
	13.839	0.59	Butylated Hydroxytoluene	128-37-0	$C_{15}H_{24}O$
	17.022	0.57	Heptadecane	629-78-7	$C_{17}H_{36}$
	17.201	0.56	Phenanthrene	85-01-8	$C_{14}H_{10}$
	14.777	0.55	Hexadecane	544-76-3	$C_{16}H_{34}$
	20.833	0.53	Phthalic acid diphenyl ester	523-31-9	$C_{22}H_{18}O_4$
	20.084	0.5	5,7-Dodecadiyn-1,12-diol	74602-32-7	$C_{12}H_{18}O_2$
	24.84	0.5	DOP	117-81-7	$C_{24}H_{38}O_4$
GC	18.831	28.13	1,2-Benzenedicarboxylic acid, bis(2-methylpropyl) ester	84-69-5	$C_{16}H_{22}O_4$
	23.966	3.25	Hexatriacontane	630-06-8	$C_{36}H_{74}$
	20.889	2.58	Tetracosane	646-31-1	CH3(CH2)22CH3
	21.38	1.76	Butane, 1-(2,2-dichloro-3,3-dimethylcyclopropyl)-	24551-91-5	$C_9H_{16}Cl_2$
	21.133	1.68	Cyclopropane, 1,1-dichloro-2,2,3-triethyl-	24551-90-4	$C_9H_{16}Cl_2$
	23.151	1.15	Triphenyl phosphate	115-86-6	$C_{18}H_{15}O_4P$
	22.555	1.06	5,5,10,10-Tetrachlorotricyclo [7.1.0.0(4,6)] decane	62990-21-0	$C_{10}H_{12}Cl_4$
	24.238	0.93	1-Penten-4-yn-3-ol, 1-phenyl-	14604-31-0	$C_{11}H_{10}O$
	19.988	0.91	Heneicosane	629-94-7	$C_{21}H_{44}$
	24.84	0.91	DOP	117-81-7	$C_{24}H_{38}O_4$
	21.216	0.89	3,7,11,15-Tetramethyl-hexadecanol, trimethylsilyl ether	645-72-7	$C_{20}H_{42}O$
	20.342	0.83	5,7-Dodecadiyn-1,12-diol	74602-32-7	$C_{12}H_{18}O_2$
	20.213	0.73	1-Heptene, 5,7,7,7-tetrachloro-	51287-99-1	$C_7H_{10}Cl_4$
	10.404	0.66	Caprolactam	105-60-2	$C_6H_{11}NO$
	22.902	0.59	1-Hexadecanol, acetate	629-70-9	$C_{18}H_{36}O_2$
	14.884	0.54	Fluorene	86-73-7	$C_{13}H_{10}$
	17.021	0.53	Heptadecane	629-78-7	$C_{17}H_{36}$
	13.656	0.52	3-Deoxyglucose	4084-27-9	$C_6H_{10}O_5$
	13.837	0.51	Butylated Hydroxytoluene	128-37-0	$C_{15}H_{24}O$
GT	18.819	41.82	1,2-Benzenedicarboxylic acid, bis(2-methylpropyl) ester	84-69-5	$C_{16}H_{22}O_4$
	20.894	2.96	Tetracosane	646-31-1	$C_{24}H_{50}$
	14.272	2.59	3-Acetoxybutyric acid	52020-45-8	$C_6H_{10}O_4$
	14.892	2.4	Fluorene	86-73-7	$C_{13}H_{10}$
	10.415	2.26	Caprolactam	105-60-2	$C_6H_{11}NO$
	13.667	1.93	3-Deoxyglucose	4084-27-9	$C_6H_{10}O_5$
	13.787	1.92	Phenol, 2,4-bis(1,1-dimethylethyl)-	96-76-4	$C_{14}H_{22}O$
	19.047	1.74	Heneicosane	629-94-7	$C_{21}H_{44}$
	29.676	1.54	Hexatriacontane	630-06-8	$C_{36}H_{74}$
	14.151	1.44	1,1,3,3-Tetramethyl-1,3-disilacyclopentane	5927-28-6	$C_7H_{16}Si_2$
	17.025	1.2	Heptadecane	629-78-7	$C_{17}H_{36}$
	14.781	1.16	Hexadecane	544-76-3	$C_{16}H_{34}$
	17.209	1.12	Phenanthrene	85-01-8	$C_{14}H_{10}$
	10.201	0.95	Benzothiazole	273-13-2	$C_6H_4N_2S$
	11.17	0.75	Cyclohexasiloxane, dodecamethyl-	540-97-6	$C_{12}H_{36}O_6Si_6$
	13.231	0.62	Biphenylene	259-79-0	$C_{12}H_8$
	12.272	0.57	Tetradecane	629-59-4	$C_{14}H_{30}$
	14.092	0.57	Dibenzofuran	132-64-9	$C_{12}H_8O$
	15.345	0.54	Dibenzofuran, 4-methyl-	7320-53-8	$C_{13}H_{10}O$
	15.509	0.54	Cyclooctasiloxane, hexadecamethyl-	556-68-3	$C_{16}H_{48}O_8Si_8$

9.244	0.53	N-Nitroso-2-methylthiazolidine	70629-19-5	$C_4H_8N_2OS$

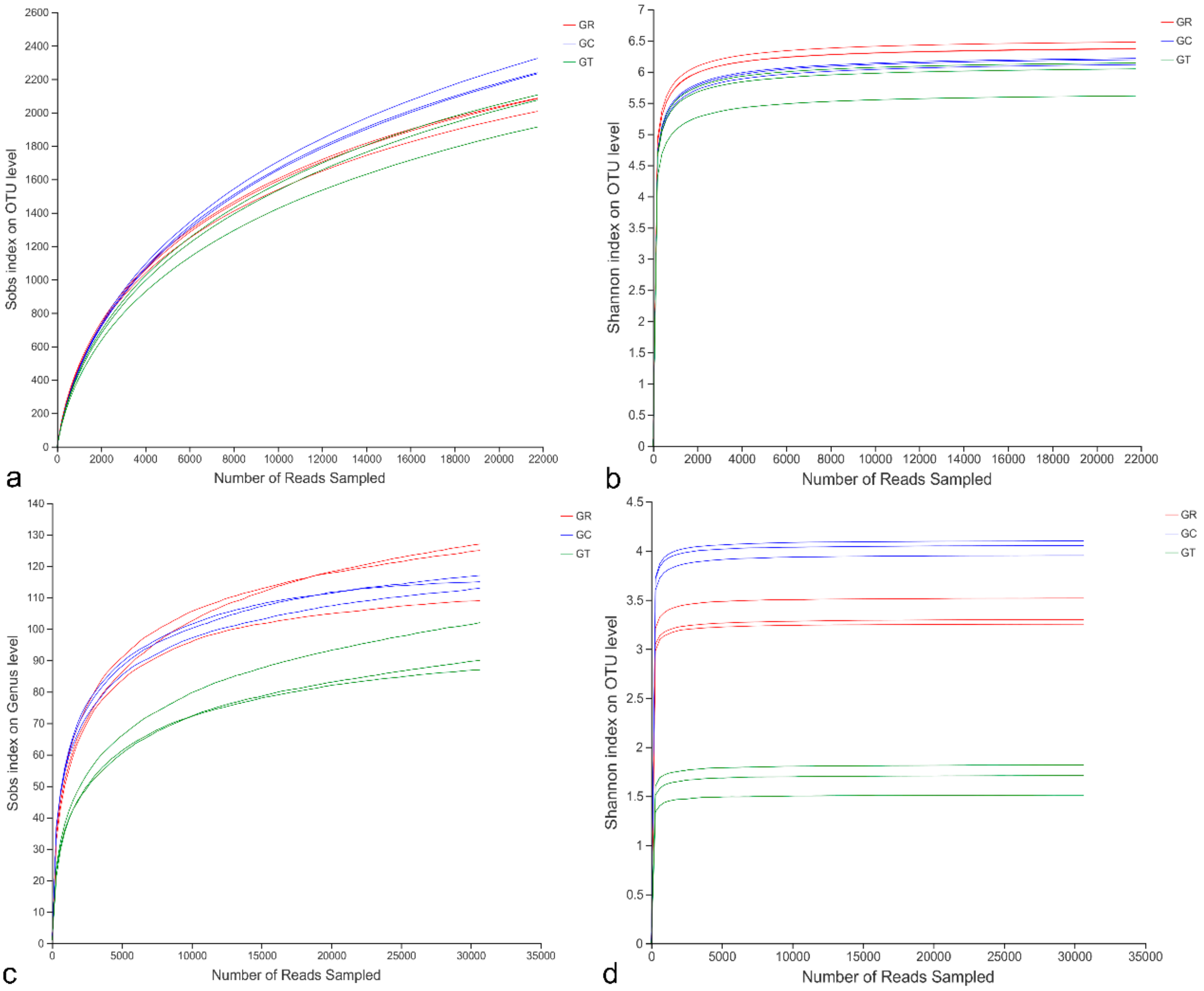

Figure S1. Dilution curve at the OTU level. Sobs index of bacteria (**a**) and fungi (**c**); Shannon index of bacteria (**b**) and fungi (**d**). GR, control replant soil in the greenhouse experiment; GC, replant soil with blank carrier in the greenhouse experiment; GT, replant soil with 6S-2 fertilizer in the greenhouse experiment.

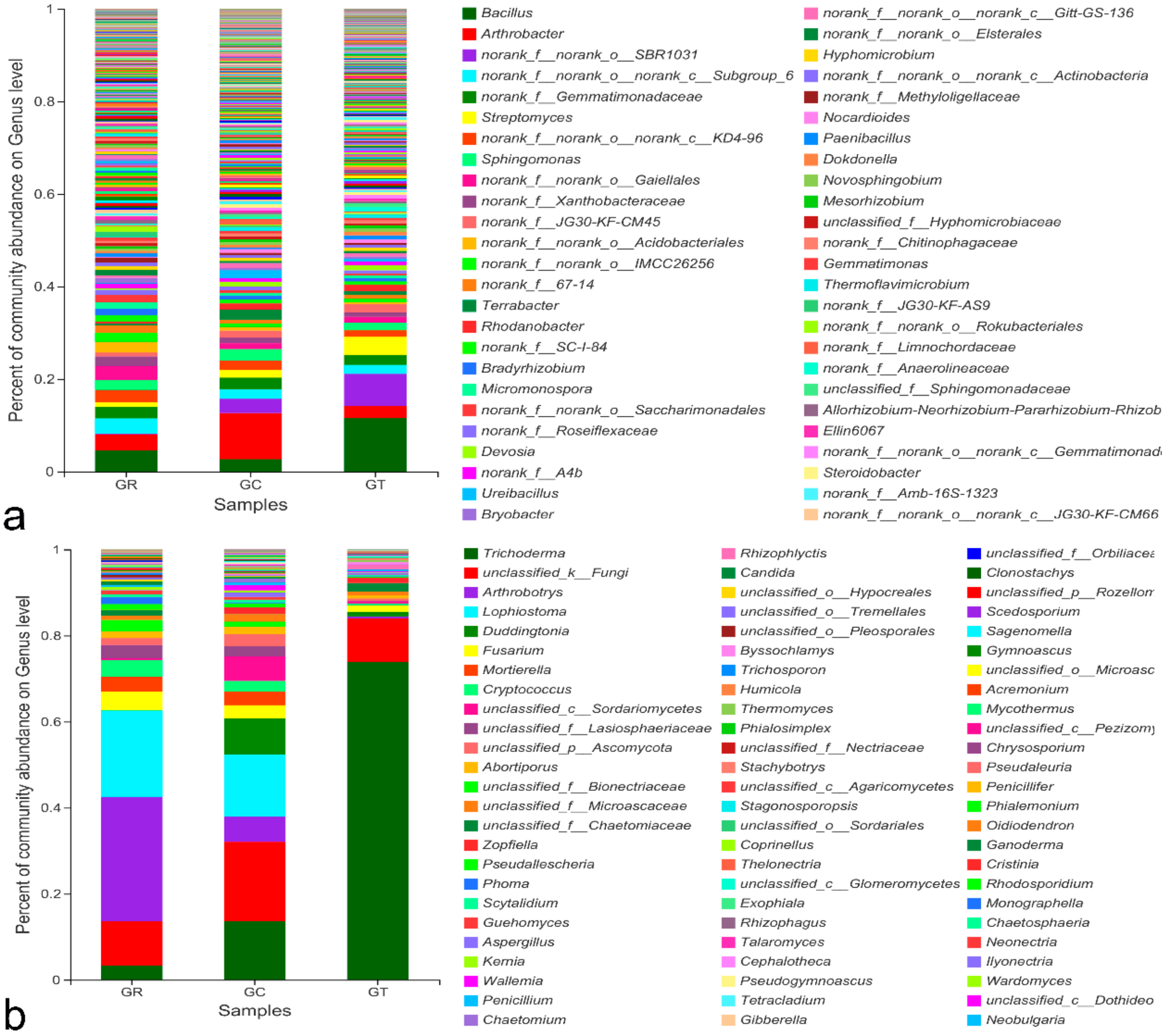

Figure S2. Species compositions of different treatments at the genus level for bacteria (**a**) and fungi (**b**). GR, control replant soil in the greenhouse experiment; GC, replant soil with blank carrier in the greenhouse experiment; GT, replant soil with 6S-2 fertilizer in the greenhouse experiment.

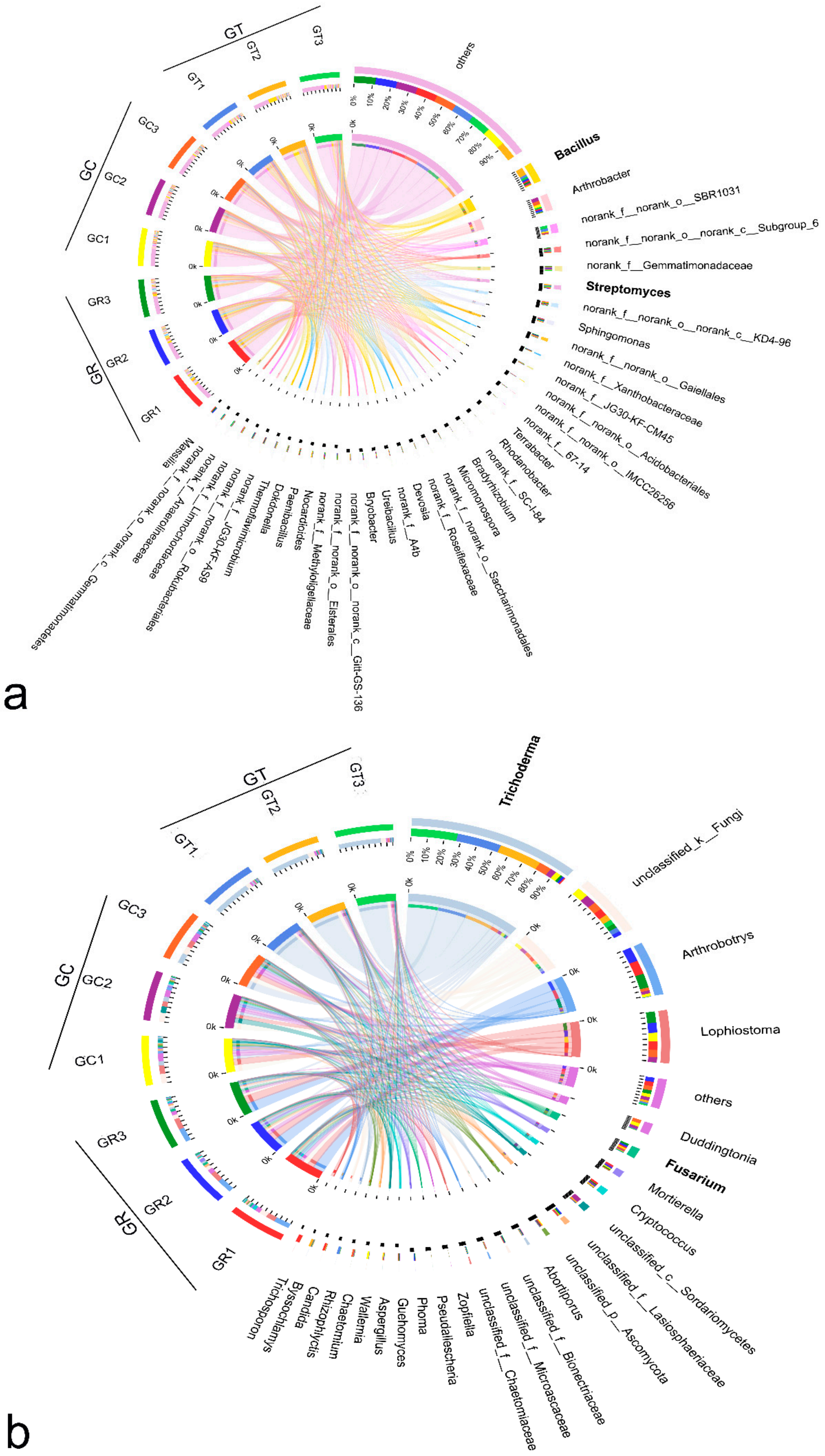

Figure S3. Circos diagrams of the relative abundance and distribution of soil bacteria (**a**) and fungi (**b**) at the genus level for different groups. The width of the bar indicates the relative abundance of the genus. GR, control replant soil in the greenhouse experiment; GC, replant soil with blank carrier in the greenhouse experiment; GT, replant soil with 6S-2 fertilizer in the greenhouse experiment.

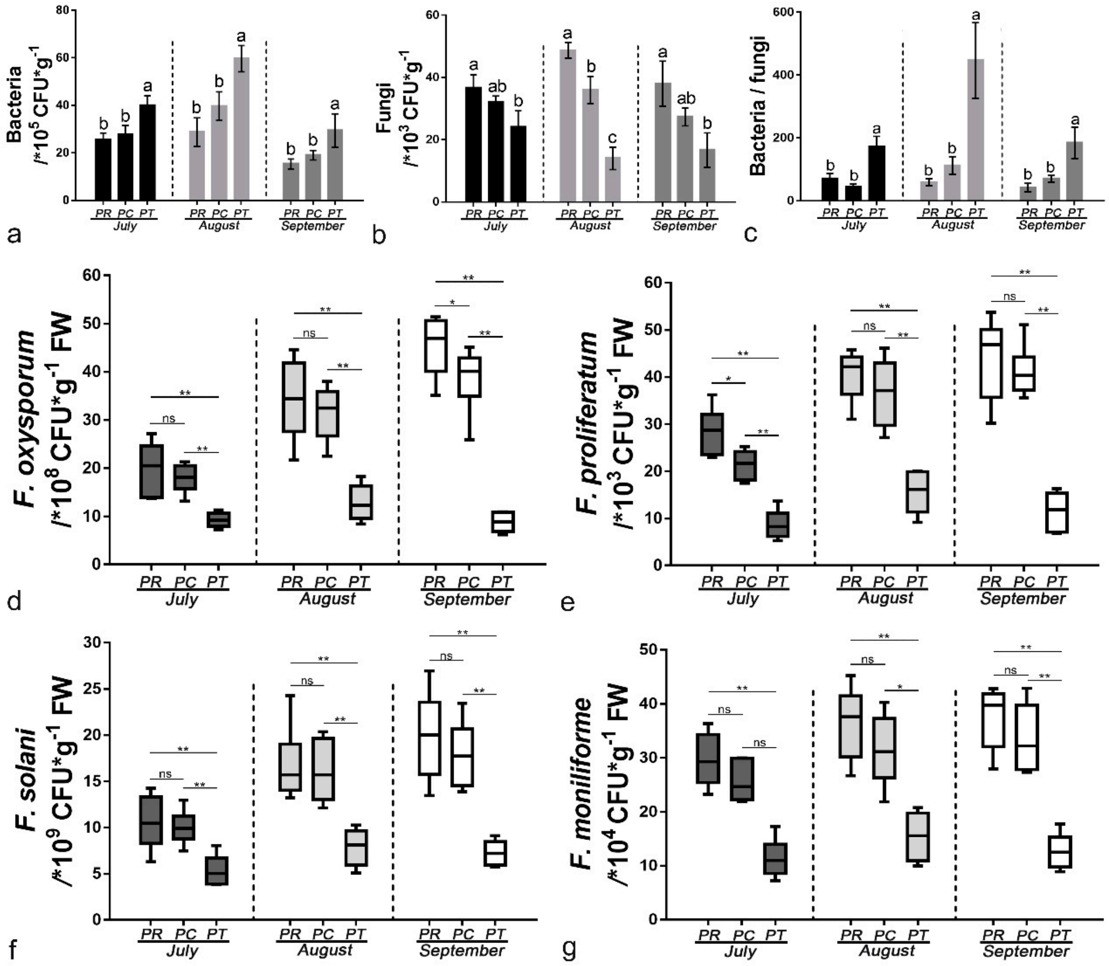

Figure S4. The number of culturable bacteria (**a**) and fungi (**b**) and the ratio of bacteria to fungi (**c**) in the pot experiment. Real-time fluorescence quantification of *F. oxysporum* (**d**), *F. proliferatum* (**e**), *F. solani* (**f**), and *F. moniliforme* (**g**) in the pot experiment. PR, control replant soil in pots; PC: replant soil with blank carrier in pots; PT: replant soil with 6S-2 fertilizer in pots. The significance of differences between groups was determined by Duncan's new multiple range test and Student's *t*-test. Different lowercase letters indicate a significant difference at $P < 0.05$ by Duncan's new multiple range test. Within a given measurement, *$P < 0.05$; **$P < 0.01$; NS, no significant difference (Student's *t*-test).

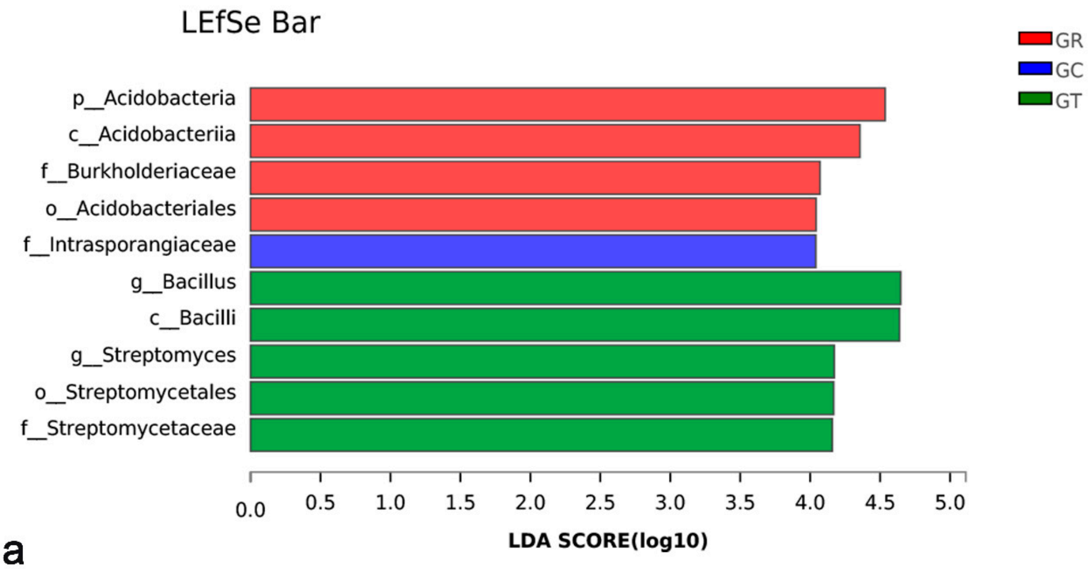

a

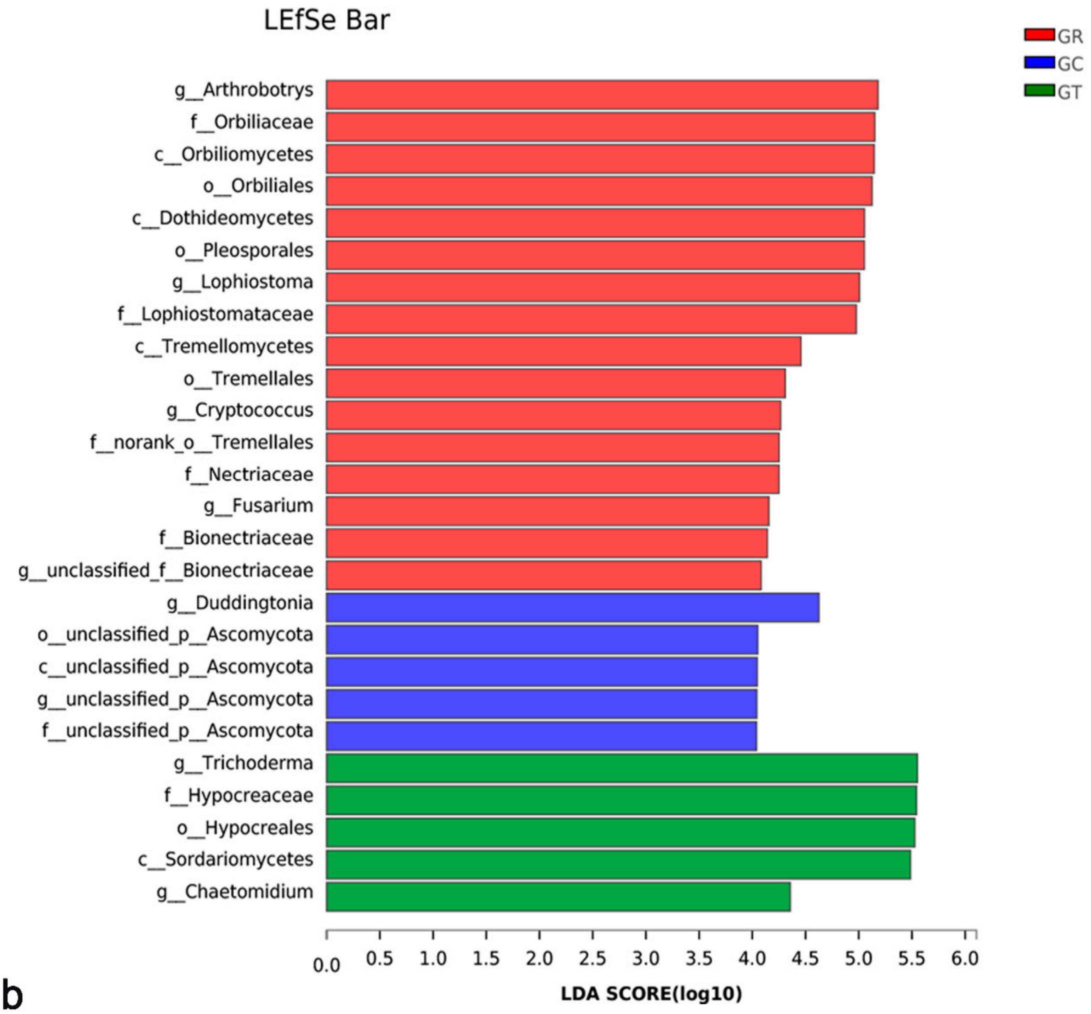

b

Figure S5. LDA scores of enriched taxa from Figure 4 (g and h). Indicator bacteria (**a**) and fungi (**b**) with LDA scores of 4 or greater in communities of the three treatment groups. GR (red), control replant soil in the greenhouse experiment; GC (blue), replant soil with blank carrier in the greenhouse experiment; GT (green), replant soil with 6S-2 fertilizer in the greenhouse experiment.

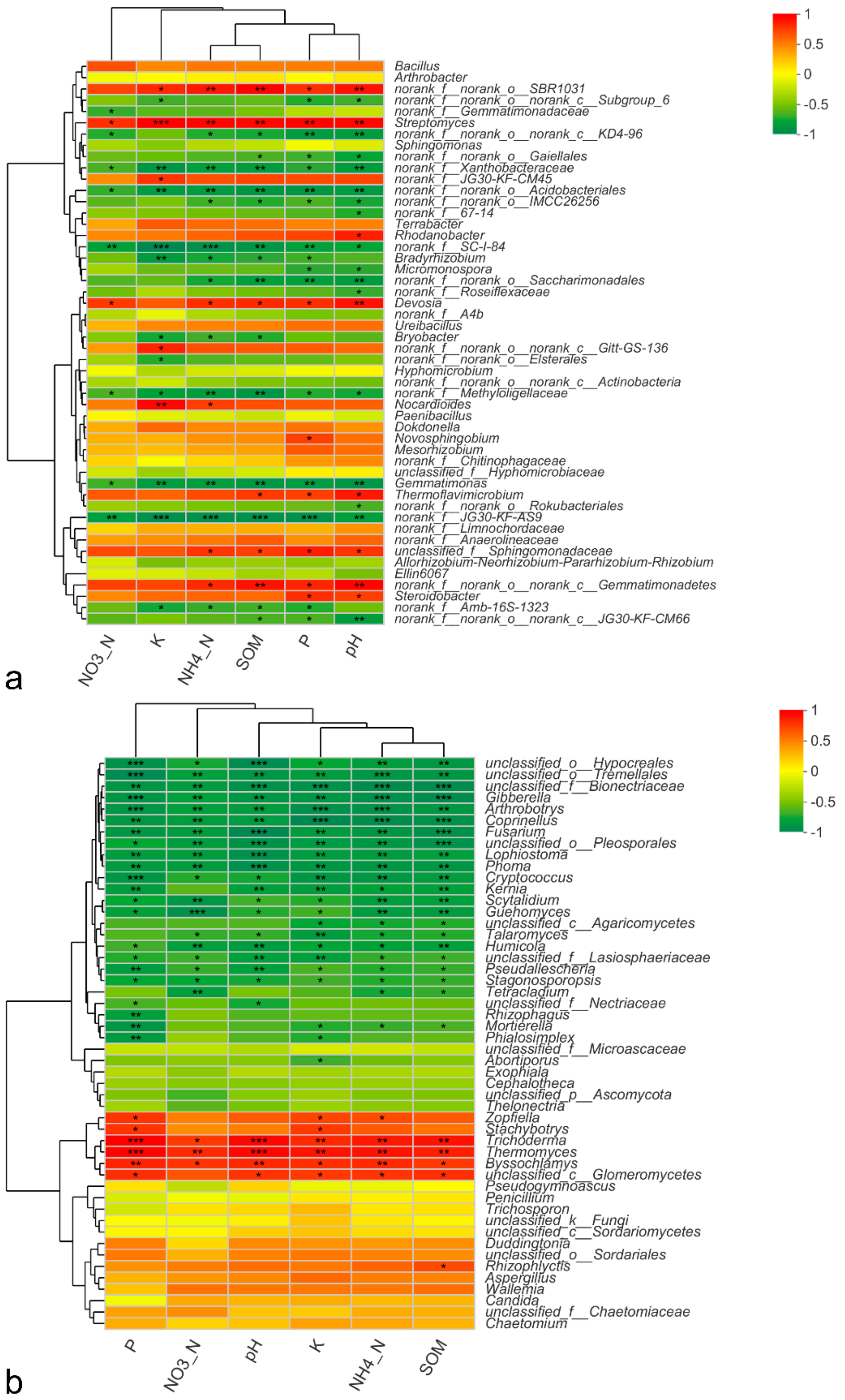

Figure S6. Correlation heatmap of the top fifty bacterial (**a**) and fungal (**b**) genera with environmental factors. The x and y axes are environmental factors and genera. The legend shows the color range of the R values. $*P < 0.05$.

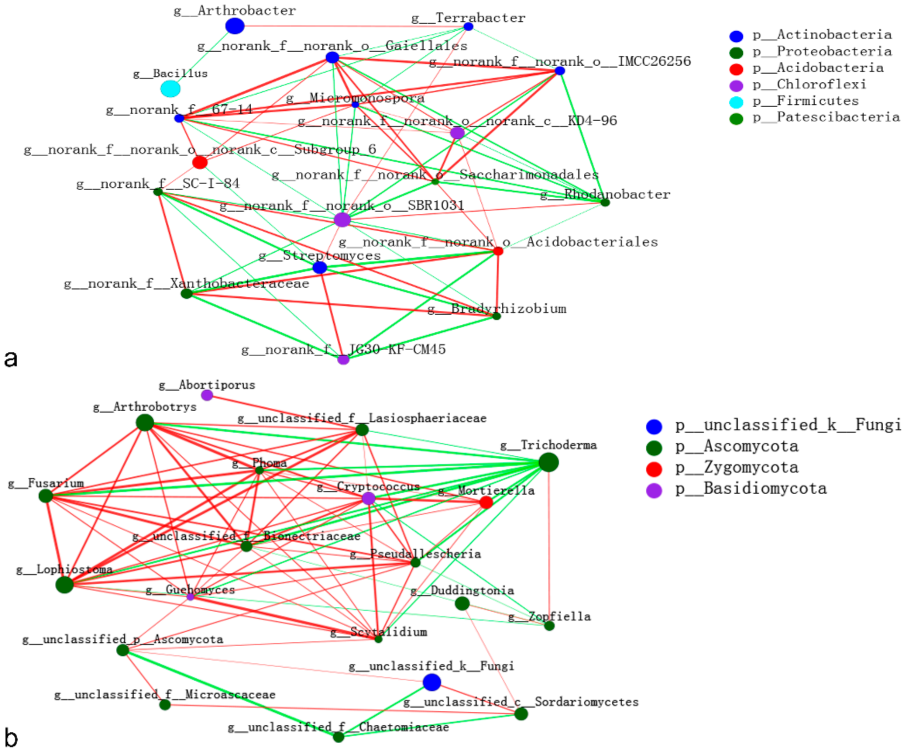

Figure S7. Single-way correlation network among the top twenty bacterial (**a**) and fungal (**b**) genera. Node size is proportional to genus abundance. Node color corresponds to family taxonomic classification. Edge colors represent positive (green) and negative (red) correlations, and the edge thickness is equivalent to the correlation value.

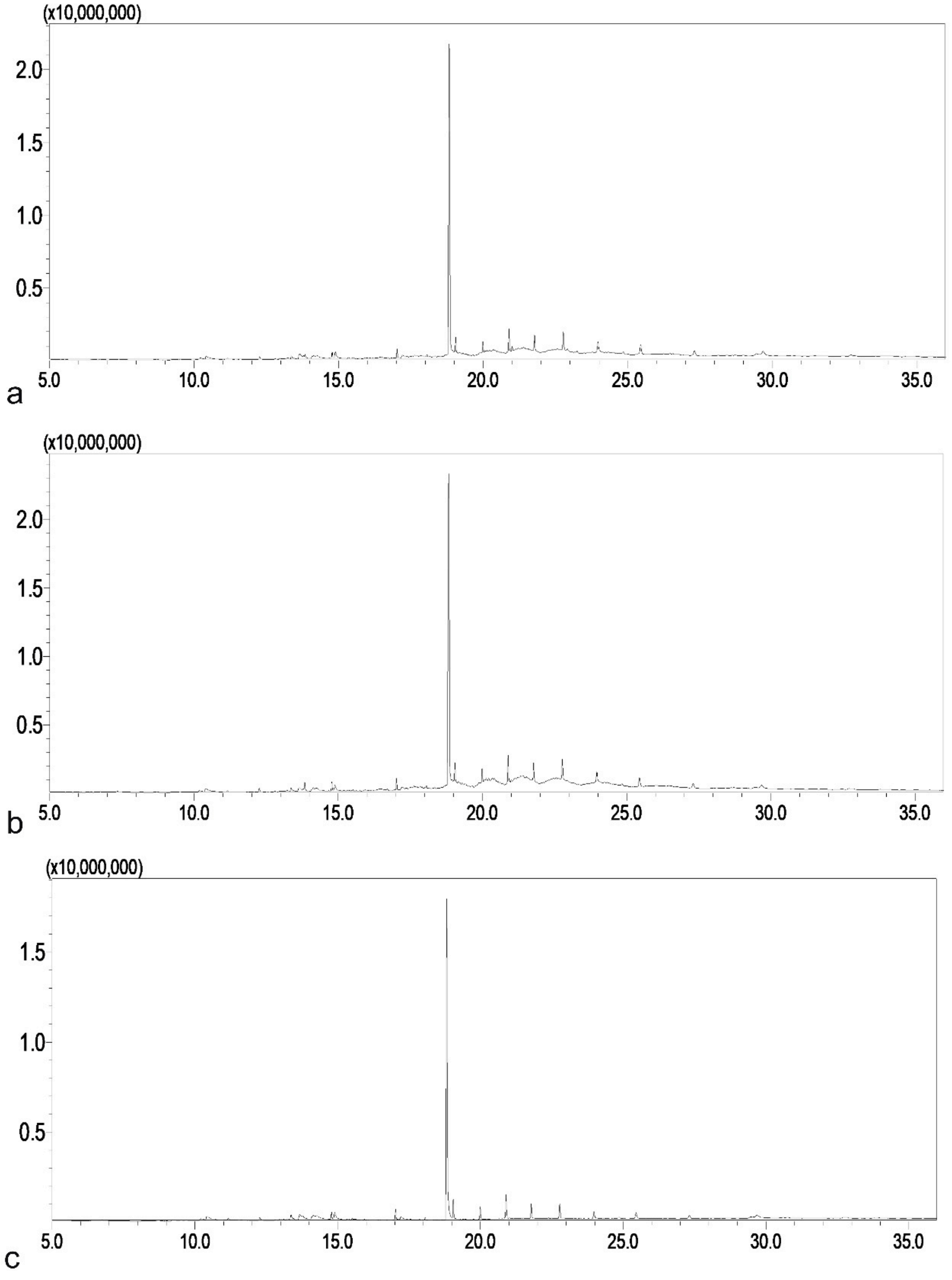

Figure S8. The spectrum of exudates from roots of *M. hupehensis* seedlings under different treatments. The x and y axes axis are time and contents.
